# CPX-351 treatment in secondary acute myeloblastic leukemia is effective and improves the feasibility of allogeneic stem cell transplantation: results of the Italian compassionate use program

**DOI:** 10.1038/s41408-020-00361-8

**Published:** 2020-10-06

**Authors:** Fabio Guolo, Luana Fianchi, Paola Minetto, Marino Clavio, Michele Gottardi, Sara Galimberti, Giuliana Rizzuto, Michela Rondoni, Giambattista Bertani, Michela Dargenio, Atto Bilio, Barbara Scappini, Patrizia Zappasodi, Anna Maria Scattolin, Francesco Grimaldi, Giuseppe Pietrantuono, Pellegrino Musto, Marco Cerrano, Stefano D’Ardia, Ernesta Audisio, Alessandro Cignetti, Crescenza Pasciolla, Francesca Pavesi, Anna Candoni, Carmela Gurreri, Monica Morselli, Caterina Alati, Nicola Fracchiolla, Giovanni Rossi, Manuela Caizzi, Fabrizio Carnevale-Schianca, Agostino Tafuri, Giuseppe Rossi, Felicetto Ferrara, Livio Pagano, Roberto Massimo Lemoli

**Affiliations:** 1 IRCCS Ospedale Policlinico San Martino, Genoa, Italy; 2 Clinic of Hematology, Department of Internal Medicine (DiMI), University of Genoa, Genoa, Italy; 3 Istituto di Ematologia, Fondazione Policlinico Universitario A. Gemelli, IRCCS, Rome, Italy; 4 Ospedale Ca’ Foncello, Treviso, Italy; 5 UO Ematologia, Dipartimento di Medicina Clinica e Sperimentale, Università di Pisa, Pisa, Italy; 6 Hematology and Bone Marrow Transplant Unit, ASST Papa Giovanni XXIII, Bergamo, Italy; 7 U.O.C. di Ematologia, Azienda Unità Sanitaria Locale della Romagna, Ravenna, Italy; 8 S.C. Ematologia, ASST Grande Ospedale Metropolitano, Niguarda Ca’ Granda Milano, Milan, Italy; 9 Ospedale Vito Fazzi, Lecce, Italy; 10 Ospedale di Bolzano, Bolzano, Italy; 11 Dipartimento di Oncologia-SODc Ematologia, Azienda Ospedaliero - Universitaria Careggi, Florence, Italy; 12 Clinica Ematologica, Fondazione IRCCS Policlinico San Matteo, Pavia, Italy; 13 Unità Operativa di Ematologia, Ospedale dell’Angelo, Mestre-Venezia, Italy; 14 Dipartimento di Medicina Clinica e Chirurgia, AOU Federico II di Napoli, Naples, Italy; 15 IRCCS Centro Oncologico della Basilicata, Rionero in Vulture, Potenza Italy; 16 Unit of Hematology and Stem Cell Transplantation, AOU Policlinico Consorziale, “Aldo Moro” University, Bari, Italy; 17 Department of Molecular Biotechnology and Health Sciences, Division of Hematology, University of Torino, Turin, Italy; 18 Institute for Cancer Research and Treatment, University of Turin-School of Medicine, Turin, Italy; 19 S.C. Ematologia2, Dipartimento di Ematologia e Oncologia, AO Città della Salute e della Scienza di Torino, Turin, Italy; 20 Divisione Universitaria di Ematologia e Terapie Cellulari, A.O. Ordine Mauriziano, Turin, Italy; 21 Haematology Unit, IRCCS Istituto Tumori “Giovanni Paolo II”, Bari, Italy; 22 Hematology and Bone Marrow Transplantation Unit, San Raffaele Scientific Institute, Milan, Italy; 23 Clinica Ematologica, Centro Trapianti e Terapie Cellulari, Azienda Sanitaria Universitaria Integrata di Udine, Udine, Italy; 24 U.O. Ematologia ed Immunologia Clinica, Azienda Ospedaliera di Padova, Padova, Italy; 25 Department of Medical and Surgical Sciences, Section of Hematology, University of Modena and Reggio Emilia, Azienda Ospedaliero Universitaria Policlinico, Modena, Italy; 26 A. O. Bianchi-Melacrino-Morelli, Reggio Calabria, Italy; 27 Oncoematologia, IRCCS Ca’ Granda Ospedale Maggiore Policlinico and University of Milan, Milan, Italy; 28 U.O. Ematologia, Casa Sollievo della Sofferenza IRCCS, San Giovanni Rotondo, Foggia Italy; 29 S.C. Ematologia Azienda Sanitaria Universitaria Integrata di Trieste, Ospedale Maggiore, Trieste, Italy; 30 Medical Oncology, Hematopoietic Stem Cells Unit, Turin Metropolitan Transplant Center, Candiolo Cancer Institute-FPO, IRCCS, Candiolo, Italy; 31 Department of Clinical and Molecular Medicine & Hematology, Sant’Andrea - University Hospital - Sapienza - University of Rome, Rome, Italy; 32 SC Ematologia e Dipartimento di Oncologia Clinica, A.O. Spedali Civili, Brescia, Italy; 33 Division of Hematology, Cardarelli Hospital, Naples, Italy

**Keywords:** Combination drug therapy, Drug development

## Abstract

Secondary acute myeloid leukemia (sAML) poorly responds to conventional treatments and allogeneic stem cell transplantation (HSCT). We evaluated toxicity and efficacy of CPX-351 in 71 elderly patients (median age 66 years) with sAML enrolled in the Italian Named (Compassionate) Use Program. Sixty days treatment-related mortality was 7% (5/71). The response rate at the end of treatment was: CR/CRi in 50/71 patients (70.4%), PR in 6/71 (8.5%), and NR in 10/71 (19.7%). After a median follow-up of 11 months relapse was observed in 10/50 patients (20%) and 12 months cumulative incidence of relapse (CIR) was 23.6%. Median duration of response was not reached. In competing risk analysis, CIR was reduced when HSCT was performed in first CR (12 months CIR of 5% and 37.4%, respectively, for patients receiving (=20) or not (=30) HSCT, *p* = 0.012). Twelve-months OS was 68.6% (median not reached). In landmark analysis, HSCT in CR1 was the only significant predictor of longer survival (12 months OS of 100 and 70.5%, for patients undergoing or not HSCT in CR1, respectively, *p* = 0.011). In conclusion, we extend to a real-life setting, the notion that CPX is an effective regimen for high risk AML patients and may improve the results of HSCT.

## Introduction

Acute myeloid leukemia (AML) is a highly heterogeneous disease including approximately one fourth of cases secondary to previous hematological disorders (sAML) or developing after chemotherapy or radiotherapy (tAML)^1–5^. sAMLs and tAMLs are more frequent in older patients and their prognosis is often worsened by the presence of adverse/complex cytogenetics, high risk molecular aberrations, and impaired performance status^6–8^. These unfavorable biologic and clinical features deeply affect the efficacy of conventional treatment that is, generally, able to induce less than 40% short-term complete remissions with poor tolerability^9,10^. Allogeneic stem cell transplantation (HSCT) is the only curative therapeutic option in this unfavorable setting^11^. However, HSCT feasibility and overall results are impaired by the low efficacy of available induction therapies, the high median age and comorbidity burden of the majority of the patients^8,11^. Recently, targeted drugs such as gemtuzumab ozogamicin (GO) and midostaurin showed promising efficacy in de novo AML but data on elderly AML patients are lacking^12,13^. CPX-351 (VYXEOS®, Jazz Pharmaceuticals) is a liposomal encapsulation of cytarabine and daunorubicin, with a molar ratio of 5:1^14,15^. A phase II study and a phase III randomized trial have shown an increased progression free survival (PFS) and overall survival (OS) in patients with sAMLs and tAMLs receiving CPX-351, compared to individuals treated with standard 3 + 7 chemotherapy. Indeed, the best outcome was observed in patients who were consolidated with HSCT^16,17^. The drug has, therefore, received Food and Drug Administration (FDA) and European Medicine Agency (EMA) approval for the treatment of adult patients with tAMLs, sAMLs, or AML with morphologic myelodysplasia-related changes (MRC–AML). Efficacy and toxicity data were subsequently confirmed in a phase 4, multicenter, single-arm, open-label early access program conducted in the United States^18^.

However, the feasibility and activity of CPX have never been evaluated outside formal clinical trials, where inclusion criteria may have produced a positive patient selection bias. Thus, the aim of the present study was to evaluate, in a cohort of patients treated according to the Italian Compassionate Use Program (CUP), the therapeutic role of CPX-351 in a real-life setting with particular focus on the outcome of patients who received HSCT in first CR.

## Material and methods

### Italian compassionate use program

The Italian compassionate use program (CUP) for CPX-351 (Vyxeos®) started on June 2018 and ended in July 2019. A total of 38 Italian Centers were included in the CUP and 33 of them actually enrolled patients. Main enrollment criteria were diagnosis of sAML or tAML according to WHO 2016 definitions and cardiac ejection fraction >50%^1^. The complete checklist for inclusion and exclusion criteria adopted in the CUP is provided in the supplementary materials section.

Following FDA and EMA approval, CUP treatment was designed and performed according to the phase III trial by Lancet et al.^17^ and consisted in:

- Induction course with a CPX-351 dose of 44 mg/m^2^ (daunorubicin 44 mg/m^2^ plus cytarabine 100 mg/m^2^) repeated on day 1, 3, and 5. A second induction with the same dose of the drug given on day 1 and 3 was allowed for patients failing to achieve at least CRi after the first induction cycle.

- Up to two consolidation courses with a CPX-351 dose of 29 mg/m^2^ (daunorubicin 29 mg/m^2^ plus cytarabine 65 mg/m^2^) repeated on day 1 and 3 were scheduled for each patient.

Allogeneic stem cell transplantation consolidation was allowed in every phase of treatment, as per internal guidelines and clinical evaluation.

Written informed consent for treatment and data collection as well as EC approval was obtained for each patient enrolled in the CUP. A total of 73 patients have been treated in 33 different Centers during the CUP. CRF forms were sent to all Centers for data collection. The study was conducted according to the Declaration of Helsinki.

Thirty-one/33 Centers (93.9%) provided complete data, accounting for 71/73 treated patients (97%).

### Diagnostic work-up

Conventional cytogenetic analysis with q-banding was performed locally in all patients and cytogenetic abnormalities were graded according to Medical Research Council Criteria^7^. Molecular work-up was performed as per local standard in all Centers and included *NPM1* and *FLT3-ITD* mutational status in almost all patients, whereas *TP53* mutations were evaluated in 37/71 patients (42.3%). Other mutations such as *IDH1*, *IDH2*, and *RUNX1* were evaluated only in a small minority of patients (data not shown). European Leukemia Net 2017 (ELN 2017) risk score was adopted for risk definition in all patients^3^.

### Response assessment, and adverse events definition

Complete response (CR) was defined by bone marrow blast count <5% with complete platelets and neutrophil count recovery, complete response with incomplete count recovery (CRi) was defined by <5% bone marrow blast count without platelet or neutrophil count recovery, whereas partial response (PR) was defined by a count recovery with a marrow blast count between 5 and 25% and reduced more than 50% from baseline, as per conventional IWG definitions^3^. Response assessment timing was chosen in each Center as per clinical standards. Minimal residual disease (MRD) assessment was not a mandatory endpoint of the study, and was performed according to internal clinical practice, following ELN suggestions^3^. *WT1*–MRD assessment was performed as recommended by ELN^19^. Flow cytometry-based MRD assessment data were available from 17/31 Centers (54.8%) for 40/71 patients (56.3%), whereas *WT1*-based MRD was available for 15/31 (48.4%) Centers for 38/71 patients (53.5%).

Adverse events during treatment were defined and graded according to common terminology criteria for adverse events (CTCAE).

### Statistical analysis

The cumulative incidence of relapse (CI) at various time points was calculated in competing risk analysis by counting nonrelapse mortality (NRM) as a competing event. Dichotomous variables were compared using the Chi-Square test or by Fisher’s exact test when necessary. Continuous variables were compared using Student’s *T*-test, or if normal distribution could not be confirmed, by Wilcoxon’s rank test. A multivariate logistic regression model was built for risk of death and risk of relapse assessment, and included only variables that reached a *p* value of at least <0.100 in early univariate analysis. Overall survival (OS) was calculated from the first day of treatment until death by any cause, or last follow-up^20^.

A separate landmark analysis was performed in order to evaluate the impact of transplantation in comparison with other variables on OS, including only patients alive and in CR after day 90. Survival curves were built using the Kaplan–Meier method, and univariate survival analysis was performed using the Log-rank test. A Cox proportional Hazard model was built for each multivariate survival analysis, including only the variables that respected proportional risk assumption. All statistical analyses, except competing risk analysis, were performed using IBMSPSS v22© running on a Debian (Linux) operating system. Competing risk analyses were performed using the Fine and Gray sub distribution relative hazard method, and compared with Gray’s test using R statistical software (www.r-project.com), running on a Debian (Linux) operating system^20^.

All two-tailed *p* values <0.05 were considered statistically significant.

## Results

### Patients

Patient’s features are summarized in Table 1.

**Table 1:** Patients characteristics.

Variable	*N* (%)
*Age*	
<70 years	51 (71.8)
>70 years	20 (28.2)
*Sex*	
Male	39 (54.9)
Female	32 (45.1)
*WBC*	
<30 × 10^9^/L	60 (84.5)
>30 × 10^9^/L	11 (15.5)
*Marrow blasts*	
<30%	22 (31.0)
>30%	49 (69.0)
*Previous HMA*	
No	54 (76.1)
Yes	17 (23.9)
*NPM1 (evaluated in 68/71, 96%)*	
Wild type	63 (92.7)
Mutated	5 (7.3)
*FLT3-ITD (evaluated in 69/71, 97%)*	
Negative	64 (92.8)
Positive	5 (7.2)
*TP53 (evaluated in 37/71, 52%)*	
Wild type	24 (64.9)
Mutated	13 (35.1)
*Karyotype*	
Favorable	3 (4.7)
Intermediate	36 (50.2)
Poor	32 (45.1)
*CPX-351 indication*	
s-AML	36 (50.2)
t-AML	22 (31.0)
MDS-related changes	13 (18.8)
*ELN 2017*	
Low/Int.	8 (11.3)
Intermediate	24 (33.8)
High	39 (54.9)

Patients were included in the CUP study from June 2018 to July 2019. One Center enrolled more than ten patients, three Centers enrolled four patients, five Centers enrolled three patients, whereas two or one patients were enrolled in 6 and 16 Centers, respectively. Median follow-up was 11 months (95% CI 10.47–11.53 months). Median age was 66 years (range 52–79).

CPX-351 was used in 22 patients (31%) for tAML, in 36 patients (51%) for sAML, evolving from MDS (31) or CMMoL (five patient), whereas in 13 patients CPX-351 was given for morphological MDS-related changes only (18%). Among 36 patients with a previous formal diagnosis of MDS or CMMoL, 17 (47%) had already received hypomethylating agents, for a median of four cycles (range 1–78) (Table 1).

62 (88%) patients had at least one comorbidity, the most frequent being hypertension (20 patients), type II diabetes (14 patients), COPD (13 patients), cardio-vascular disease (nine patients), hypothyroidism (seven patients), NASH (three patients). 25 (35%) patients had been previously diagnosed with another neoplasia, 23 of them had already received chemo or radiotherapy. Notably, four patients had prior autologous hematopoietic stem cell transplantation for low grade lymphoma (n. two patients) and multiple myeloma (n. two patients). Six patients (9%) presented with ECOG 3–4 upon enrollment.

Median WBC count at diagnosis was 3.3 × 10^9^/L (range 0.4–213). Karyotype was abnormal in 40 patients (56.3%). The most frequent abnormalities were complex karyotype (18 patients), deletion of chromosome 7, (eight patients), del(5q) (seven patients). *NPM1* mutations were found 5/68 patients (7.4%), whereas *FLT3-ITD* mutations occurred in 5/69 patients (7.2%). None of the patient had concomitant *FLT3-ITD* and *NPM1* mutation. *TP53* mutations were found in 13/37 assessed patients (35.1%). In almost all cases (12/13), the mutation was found in the context of a complex karyotype.

### Treatment overview

All 71 patients received CPX-351 first induction. Three patients (4.2%) died before response assessment, so that 68 patients were eligible for further CPX-351 therapy as per protocol.

**Fig. 1:**
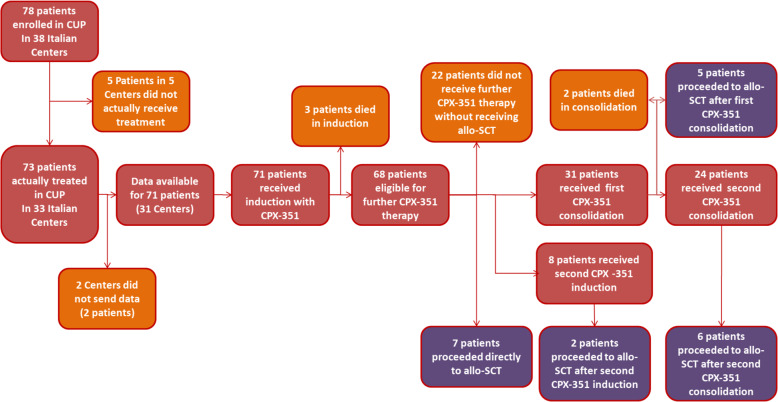
Treatment overwiev. Patients enrollment and treatment in Compassionate Use Program.

Seven (11%) patients proceeded directly to HSCT after first CPX-351 induction, whereas 22 patients did not receive further treatment, mostly for lack of response or disease progression (n. 12), impossibility to get CPX-351 due to termination CUP and lack of commercial drug (n. 6), patient refusal (n. 4). Therefore, 39 proceeded with CUP treatment program; 8/68 (12%) patients received second CPX-Induction, whereas 31/68 (46%) patients proceeded with first CPX-351 consolidation course with 24 (77%) of them receiving also the second course. Two patients died during consolidation therapy and 60 days treatment-related mortality was, therefore, 5/71 (7%). 5 out of 31 patients (16%) proceeded to HSCT after the first CPX-351 consolidation cycle, whereas six patients received HSCT after the second consolidation course and two after second induction. Two more patients received HSCT: one with progressive disease after loss of CR previously achieved with CPX-351 and one as consolidation of CR achieved with second line therapy after failure of CPX-351 induction. Main reason for not performing HSCT were poor performance status, comorbidities, and patient refusal. A detailed overview of patient flow in the CUP program is provided in Fig. 1.

### Treatment toxicities

Mortality rate following first CPX-351 course was 4.2% (3/71). 57 (80.3%) patients experienced grade >1 adverse events (AEs) during induction.

Most of the AEs were infections, with fever of unknown origin (FUO) in 20/71 (28%), sepsis in 20/71 (28%), pneumonia in eight patients (11.3%), including two *Pneumocistis jirovecii*-related pneumonia (PCP), invasive fungal infections in three patients (4.2%). Mucositis was reported in five patients (7%), whereas a self-resolving diffuse skin rash was observed in 18/71 patients (25.4%). Four patients experienced alopecia (5.6%). Most of the AEs were easily manageable and resolved completely. Median time to neutrophil >0.5 × 10^9^/L and platelet >25 × 10^9^/L recovery was 38 (range 12–60) and 28 (range 12–60) days, respectively.

Median time from first day of induction cycle and first day of second cycle was 55 days (range 33–101). Second cycle therapy-related mortality was 5.1% (2/39). Grade >1 AEs were reported in 25/39 patients (64.1%) during second cycle, including FUO in nine patients (23.7%), sepsis in 3 (7.7%), pneumonia in 3 (7.7%), including 1 PCP pneumonia, mucositis in 2 (2.8%), and diffuse skin rash in 8 (20.5%).

Overall, 60 days treatment-related mortality was 5/71 (7%), due to uncontrolled infections (n. three patients) or bleeding (n. two patients).

### Response evaluation

Response to first induction was evaluated in 68/71 surviving patients after a median of 36 days from first day of CPX-351 administration. CR was observed in 38/71 patients (53.5%) whereas CRi and PR were reported in 8/71 (11.3%) and 6/71 patients (8.5%), respectively. Sixteen patients did not respond (NR) (22.5%) and three patients died before response evaluation (4.2%), two because of uncontrolled CNS bleeding and one because of severe pneumonia. Among the 40 patients undergoing MFC MRD analysis, MRD negative CR was reached in 15 patients (37.5%). 21 (53.8%) of the 38 patients evaluated with *WT1*-based MRD assessment achieved *WT1*–MRD negativity.

At the end of CPX treatment (EOT) 50/71 (70.4%) patients were in CR, 6/71 (8.5%) in PR and 10/71 (14.1%) were refractory, whereas treatment-related mortality was 7% (five patients).

Specifically, one CR patient died during first consolidation because of uncontrolled infection and one CRi patient lost response while the other 44 maintained CR/CRi at EOT. Among PR patients, one died after second cycle because of uncontrolled infection, two achieved CR, four maintained PR, and one lost response at EOT. Six of the 14 NR patients received second induction: 4 and 2 of them achieved CR and PR, respectively, at EOT.

Response probability was not influenced by any of the analyzed variables (Table 2). In particular, the response rate was not lower in patients who had failed previous HMA for MDS (CR rate of 10/17 and 40/54, for patients with or without previous HMA treatment, respectively, *p* = 0.361), in patients harboring *TP53* mutations (CR rate of 10/13 and 18/24 for patients with or without *TP53* mutations, respectively, *p* = 1.000), in patients with high-risk disease according to ELN 2017 (CR rate of 25/32 and 25/39, for patients with low/intermediate vs. high-risk disease, respectively, *p* = 0.291). CR rates reported in patients with high risk cytogenetic features (CR rate 19/32, 59.4%), such as complex karyotype (CR rate of 11/18, 61.5%) or deletion of chromosome 5 or 7 (CR rate 8/15, 53.5%) were not significantly different from those observed in patients with either normal or nonhigh risk cytogenetic (CR rate of 31/37, *p* = 0.074). Age at diagnosis did not impact on CR probability both if evaluated continuously or with any chosen cut-off. Multivariate logistic regression analysis showed that only high risk cytogenetics and marrow blast count above 30% had a borderline, nonsignificant, impact on response probability (*p* = 0.057 and 0.062, respectively).

**Table 2:** Complete remission analysis.

Variable	*N* (%)	CR–CRi (%)	*p*-value (univariate)	Hazard ratio (95% CI)*	*p*-value (multiv.)
Overall	71 (100%)	50 (70.4%)	–	–	–
*Age*					
<70 years	51 (71.8)	37 (72.5)	0.531	0.901 (0.639–1.271)	–
>70 years	20 (28.2)	13 (65.0)			
*Sex*					
Male	39 (54.9)	29 (74.4)	0.446	0.821 (0.495–1.363)	–
Female	32 (45.1)	21 (65.6)			
*WBC*					
<30 × 10^9^/L	60 (84.5)	42 (70.0)	1.000	1.020 (0.825–1.262)	–
>30 × 10^9^/L	11 (15.5)	8 (72.7)			
*Marrow blasts*					
<30%	22 (31.0)	19 (86.4)	0.055	0.376 (0.124–1.136)	0.062
>30%	49 (69.0)	31 (63.3)			
*Previous HMA*					
No	54 (76.1)	40 (74.1)	0.361	0.833 (0.598–1.162)	–
Yes	17 (23.9)	10 (58.8)			
*NPM1*					
Wild type	63 (92.6)	42 (66.7)	0.176	1.194 (1.012–1.235)	0.150
Mutated	5 (7.4)	5 (100)			
*FLT3-ITD*					
Negative	64 (92.8)	45 (70.3)	1.000	0.965 (0.825–1.119)	–
Positive	5 (7.2)	3 (60.0)			
*TP53*					
Wild type	24 (64.9)	18 (75.0)	1.000	1.037 (0.605–1.776)	–
Mutated	13 (35.1)	10 (76.9)			
*Karyotype*					
Fav./Int.	39 (54.9)	31 (79.5)	0.074	0.614 (0.342–1.105)	0.057
Poor	32 (45.1)	19 (59.4)			
*Therapy related*					
No	49 (69.0)	37 (75.5)	0.260	0.752 (0.515–1.158)	–
Yes	22 (31.0)	13 (59.1)			
*ELN 2017*					
Low/Int.	32 (45.1)	25 (78.1)	0.296	0.667 (0.343–1.297)	–
High	39 (54.9)	25 (64.1)			

*Hazard ratio calculation refers to the first row of each variable.

Detailed analysis of CR probability is shown in Table 2.

### Cumulative incidence of relapse

After a median follow-up of 11 months (95% CI 10.47–11.53 months), relapse was observed in 10/50 responding patients (20%) and 12 months CIR was 23.6%, whereas median duration of response was not reached.

**Fig. 2:**
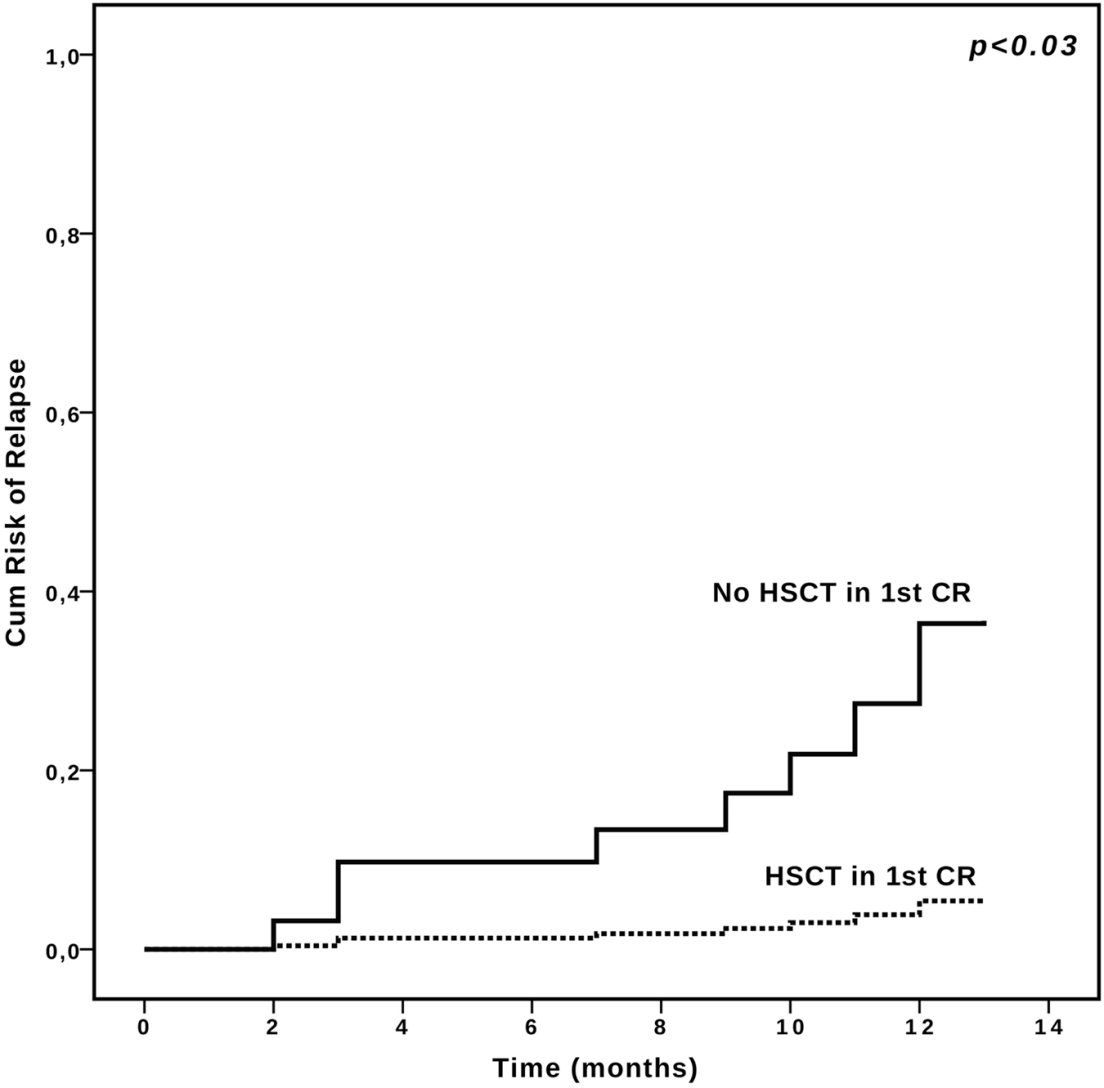
Relapse Risk in responding patients according to transplantation. Cumulative risk of relapse in patients achieving complete remission (CR), receiving or not hematopoietic stem cell transplantation (HSCT).

In competing risk analysis, a lower CIR was observed only when consolidation therapy with HSCT was performed in first CR after CPX-351 therapy, (12 months CIR of 5% and 37.4%, respectively, for patients receiving or not HSCT, respectively, *p* = 0.012, Fig. 2). A trend towards reduced CIR was observed among patients with MFC MRD negative CR, however without reaching statistical significance (12 months CIR of 11.1% and 36.7%, respectively, for patients with MRD negative or positive CR, *p* = 0.151). *WT1*-based MRD analysis led to superimposable results (data not shown).

Multivariate analysis confirmed that HSCT consolidation was the only independent predictor of lower CIR (*p* < 0.05, data not shown).

### Overall survival

Overall, 21 patients died (29.6%), mostly because of refractory disease (*n* = 16).

**Fig. 3:**
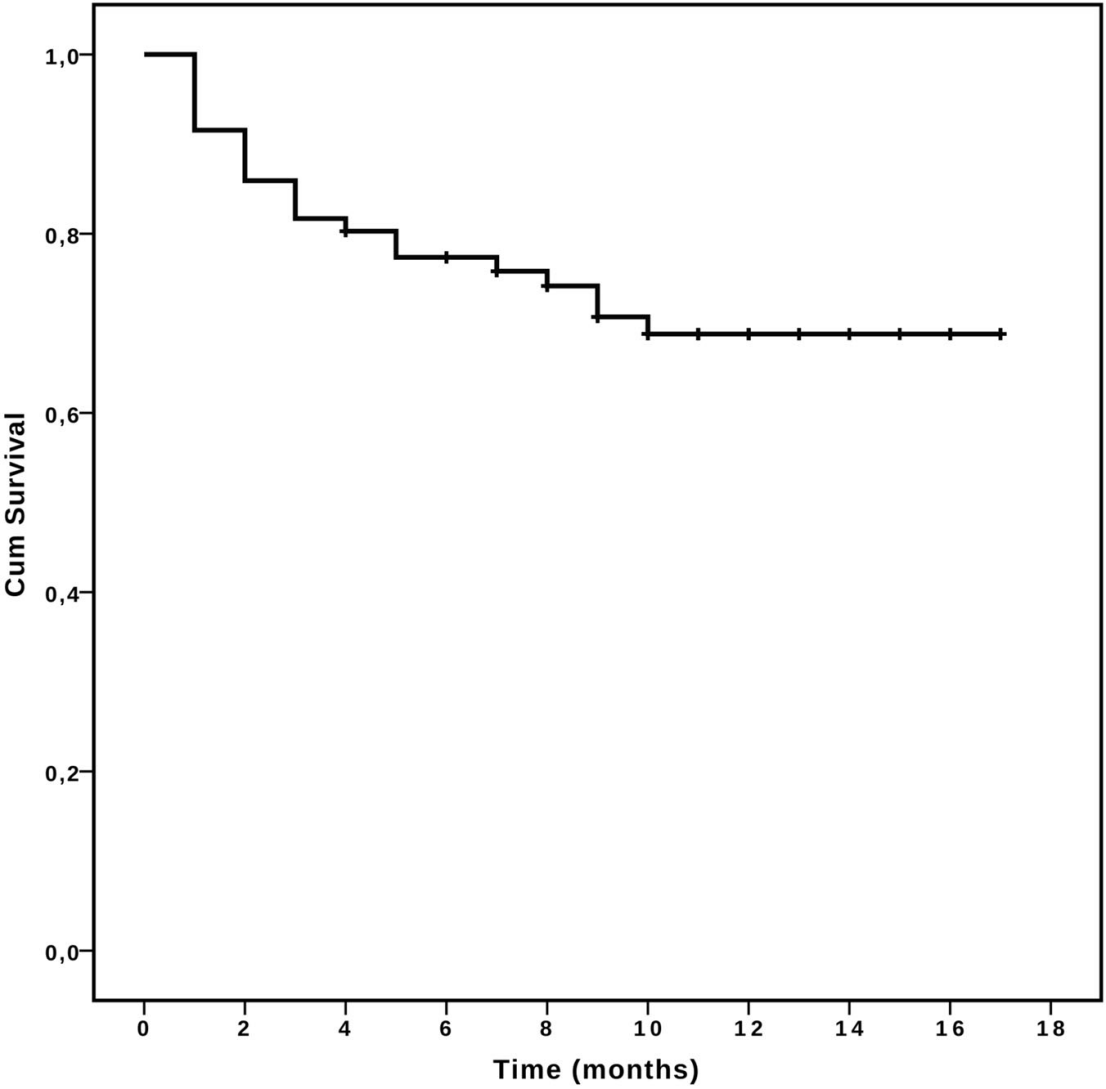
Overall Survival. Overall Survival in the whole cohort from the time of enrollment in Compassionate Use Program.

Twelve-months OS was 68.6% (median not reached, Fig. 3). In univariate analysis, survival probability was affected only by cytogenetic risk (12 months OS of 78.6 and 56.6%, for patients with favorable/intermediate and poor risk karyotype, respectively, *p* < 0.05). Failure of previous HMA therapy, ELN 2017 risk score, as well as presence of *TP53* mutation did not impact on survival (Table 3). Notably, MRD status after cycle 1 did not affect survival (12 months OS of 71.1 % vs. 84.0% for MRD-negative and MRD-positive patients, respectively, *p* = 0.414). *WT1*-based MRD analysis led to similar results (data not shown).

**Table 3:** Overall survival analysis.

Variable	Alive (%)	12-month OS (%)	Median OS	*p*-value (univariate)	*p*-value (multiv.)
Overall	50/71(70.4)	68.6	NR	–	–
*Age*					
<70 years	38/51 (78.5)	73.0	NR	0.241	–
>70 years	12/20 (60.0)	58.3	NR		
*Sex*					
Male	27/39 (69.2)	68.0	NR	0.877	–
Female	23/32 (71.9)	69.3	NR		
*WBC*					
<30 × 10^9^/L	43/60 (71.7)	70.0	NR	0.490	–
>30 × 10^9^/L	7/11 (63.6)	62.3	NR		
*Marrow blasts*					
<30%	17/22 (77.3)	76.7	NR	0.371	–
>30%	33/49 (67.3)	64.9	NR		
*Previous HMA*					
No	16/54 (70.4)	68.9	NR	0.945	–
Yes	15/17 (70.6)	69.1	NR		
*NPM1*					
Wild type	43/63 (68.3)	66.6	NR	0.162	–
Mutated	5/5 (100)	100	NR		
*FLT3-ITD*					
Negative	45/64 (70.3)	68.3	NR	0.570	–
Positive	3/5 (60.0)	60.0	NR		
*TP53*					
Wild type	20/24 (83.3)	83.1	NR	0.081	0.570
Mutated	7/13 (53.8)	51.9	NR		
*Karyotype*					
Fav./Int.	31/39 (79.5)	78.6	NR	0.049	0.051
Poor	19/32 (59.4)	56.6	NR		
*Therapy related*					
No	35/49 (71.4)	68.7	NR	0.717	–
Yes	15/22 (68.2)	68.2	NR		
*ELN 2017*					
Low/Int.	26/32 (88.9)	79.9	NR	0.073	0.071
High	24/39 (64.7)	59.5	NR		
*MRD TP1*					
Negative	11/15 (73.3)	71.1	NR	0.414	–
Positive	21/25 (84.0)	84.0	NR		

Multivariate OS analysis confirmed that karyotype was the only independent predictor of survival, however with only borderline significance (*p* = 0.051). Detailed OS analysis is provided in Table 3.

In order to assess the impact of HSCT in first CR and the correlation with the other variables, a landmark model was applied, including only patients alive and in CR at day 90. A total of 20/50 (40%) patients achieving CR with CPX-351 underwent HSCT consolidation (Fig. 1). Median age of patients submitted to HSCT was 65.5 years (range 54–73). Four of them (20%) had failed previous HMA for MDS, four had t-AML (20%), 13 had high risk disease according to ELN 2017 (65%). Cytogenetic was unfavorable in nine patients (45%), with deletion of chromosome 5 or 7 in 6 and complex karyotype in 3. Among the 13 patients tested, 3 (23.1%) had a *TP53* mutation.

**Fig. 4:**
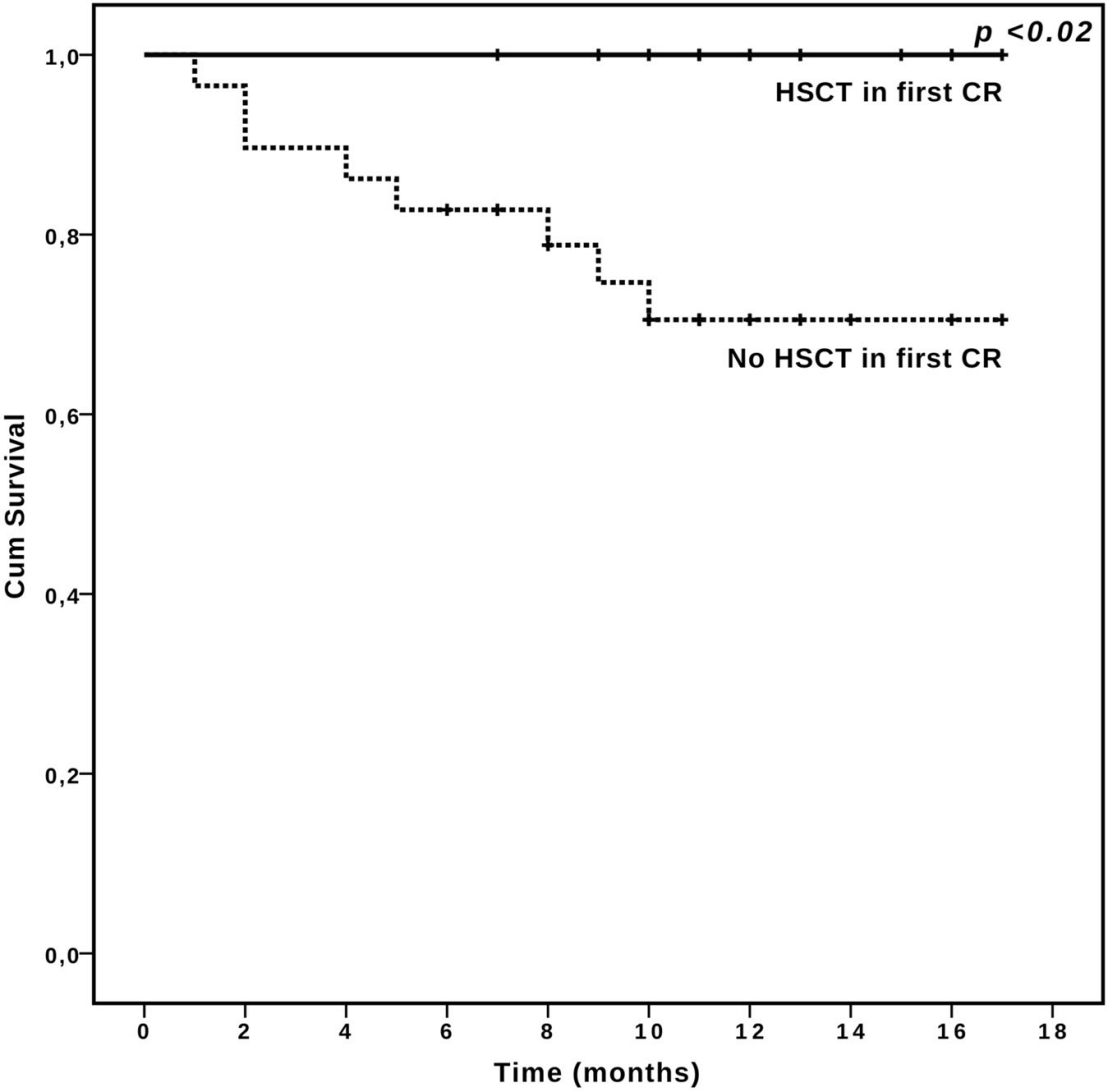
Overall Survival in responding patients according to transplantation. Landmark Overall Survival analysis in patients alive and in complete remission (CR) at day 90, receiving or not hematopoietic stem cell transplantation (HSCT).

In landmark analysis, HSCT performed in first CR after CPX-351 was the only significant predictor of longer survival (12 months OS of 100 and 70.5%, for patients receiving or not HSCT in CR1, respectively, *p* = 0.011, Fig. 4). None of the other variables affected survival according to landmark analysis.

Multivariate analysis confirmed that HSCT in first CR was the only independent predictor for OS (*p* < 0.05). Detailed overview of the landmark model is provided in supplementary material (Supplemental Table 1).

## Discussion

As the outcome of sAML and tAML is unsatisfactory with conventional chemotherapy, and the probability of performing HSCT is low, the results produced by CPX-351 represent a remarkable improvement in the treatment of these diseases^8–10,16,17,21–23^. Here, in a real life multicenter setting, we confirm the observation of the low toxicity and the high efficacy of CPX-351, and report an increased number of patients undergoing transplant^16–18^.

Indeed, despite the prolonged hematological recovery after CPX-351 induction, related to the extended drug exposure^15,24,25^, the incidence of mucositis, severe infectious complications and the mortality rate at 30 and 60 days were lower than those observed after conventional intensive chemotherapy^2,9^, and comparable to what reported in previous CPX trials^16–18^. It should be noted that our patients had multiple comorbidities, including concomitant active neoplasms, that in many cases would have precluded their enrollment into phase II–III clinical trials with CPX-351^16–18^.

Response to treatment was comparable to previous reports^16–18^. Notably CR probability was not affected by any of the most relevant prognostic factors, such as ELN 2017 risk^3^ and unfavorable cytogenetics^7^. Interestingly even the presence of *TP53* mutation in the context of a complex karyotype did not impact on the CR rate. This is in contrast with a previous paper showing the detrimental influence of *TP53* mutations on CR/CRi rate in patients treated with CPX-351 induction^26^, but consistent with more recent data from a French Group^27^, thus suggesting the need of further studies in this setting. In addition, CR rate was not lower among patients progressed under hypomethylating therapy for MDS. This observation, if confirmed in larger series of patients, may have a great clinical value as many trials have reported the lack of activity of conventional treatment after failure of azacitidine or decitabine^28,29^.

As expected, the duration of response was shorter in patients who cannot proceed to HSCT consolidation, but still satisfactory given the overall poor prognosis of our cohort. The best outcome was observed in patients who promptly underwent transplant after achieving CR. In this view, Lancet et al. showed that the outcome of patients who were transplanted following CPX therapy was significantly better compared to that observed in patients undergoing transplant after conventional 3 + 7 therapy, due to a lower transplant related mortality (TRM) and a reduced post-transplant relapse rate^17^. The transplant rate in our series (40% among patients achieving CR after CPX-351, 28.2% overall) was slightly higher than that reported in phase II and III trials^16,17^ and double than that reported in the extended access program by Roboz et al.^18^. The growing awareness of the lower TRM of HSCT after CPX-351 induction probably led Italian hematologists to a more aggressive transplant policy including the use of alternative donors. Despite the high median age of transplanted patients, no transplant related deaths have been so far reported. It might be speculated that the reduction of TRM was related to the lower extra hematological toxicity during CPX-351 induction and consolidation courses. It is unclear whether the reduced post-transplant relapse rate with CPX-351 might be due to a deeper leukemic cells clearance prior to transplant, as MRD was not evaluated in the phase II and III trials^16,17^. Considering that pretransplant MRD has a strong impact on postHSCT relapse risk^30,31^, further trials with CPX-351 should include evaluation of MRD before HSCT. In our study, we evaluated MRD with either MFC or *WT1* levels showing that near 50% of complete remissions were MRD negative. Preliminary data from a French compassionate program reported a similar rate of MRD negativity evaluated with next generation sequencing^26^. In our study, however, albeit showing a trend toward reduced relapse risk, MRD-negativity did not result in better clinical outcome possibly due to the relatively low number of patients.

An alternative explanation is that MRD assessment after the first CPX course may not represent the most informative time point. Indeed, Buccisano et al. showed that MRD analysis after the second chemotherapy course had the highest prognostic value^32^.

In conclusion, Italian CUP experience confirms that CPX-351 is an effective regimen for high risk AML patients treated with a curative aim. CPX-351 can induce good quality remissions with acceptable toxicity in the majority of patients, and increases results of HSCT, through a reduction of TRM and post-transplant relapse rate. Furthermore, the lower incidence of severe complications expected with CPX-351, compared to conventional treatment, may reasonably increase the number of elderly patients receiving intensive induction and HSCT consolidation. For frail subjects, CPX-351 cannot be recommended as it induces the long-lasting aplasia requiring prolonged hospitalizations. Further studies are needed to investigate the potential role of CPX-351 in combination with other innovative therapeutic agents^33^ and to identify the factors predicting response^34^.

## Supplementary information

Supplemental Material

Reproducibility Checklist
