# Supplementary material for: CPX-351 treatment in secondary acute myeloblastic leukemia is effective and improves the feasibility of allogeneic stem cell transplantation: results of the Italian compassionate use program
Source: Blood Cancer J. 2020 Oct 6;10(10):96. doi: 10.1038/s41408-020-00361-8 (PMC7538937; doi:10.1038/s41408-020-00361-8)
Supplement: Supplementary file 1 — Supplemental Material [file 41408_2020_361_MOESM1_ESM.docx]

**Supplementary materials**

Main CUP inclusion criteria were:

- Ability to understand and voluntarily give informed consent
- Age ≥18 years (no upper age limit)
- Pathological diagnosis of AML according to WHO criteria [CIT]
- Confirmation of:

Therapy-related AML (tAML) with documented history of prior cytotoxic therapy or ionizing radiotherapy for an unrelated disease

AML with a history of MDS (sAML), with a bone marrow documentation of prior MDS

AML with a history of Chronic Myelo-monocitic Leukemia (CMMoL)

De novo AML with karyotypic abnormalities characteristic of MDS [CIT]

Morphological alterations consistent with MDS-related changes [CIT]

- Cardiac ejection fraction >=50%

Main CUP exclusion criteria were:

- History of previous myeloproliferative neoplasm other than CMMoL
- Recognized cytogenetically favorable-risk AML at the time of treatment
- Uncontrolled secondary malignancy
- Prior treatment for AML other than hydroxyurea
- History of Wilson’s disease
- Class III or IV New York Heart Association myocardial impairment

**Supplemental Table 1:**

**OVERALL SURVIVAL LANDMARK ANALYSIS ON PATIENTS ALIVE AND IN CR AFTER 2 CYCLES**

| Variable |  | Alive (%) | 12-month OS  (%) | Median OS | p-value (univariate) | p-value  (multiv.) |
| --- | --- | --- | --- | --- | --- | --- |
| OVERALL |  | **41/50 (82.0)** | **81.2** | **NR** | **-** | **-** |
| Age | <70 years | 32/37 (86.5) | 86.0 | NR | 0.173 | - |
|  | >70 Years | 9/13 (69.2) | 66.7 | NR |  |  |
| Sex | Male | 24/29 (82.8) | 82.2 | NR | 0.807 | - |
|  | Female | 17/21 (81.0) | 79.2 | NR |  |  |
| WBC | <30x10^9^/L | 35/42 (83.3) | 82.4 | NR | 0.537 | - |
|  | >30x10^9^/L | 6/8 (75.0) | 75.0 | NR |  |  |
| Marrow Blasts | <30% | 15/19 (78.9) | 78.6 | NR | 0.664 | - |
|  | >30% | 26/31 (83.9) | 82.6 | NR |  |  |
| Previous HMA | No | 33/40 (82.5) | 81.4 | NR | 0.853 | - |
|  | Yes | 8/10 (80.0) | 80.0 | NR |  |  |
| NPM1 | Wild Type | 34/42 (68.3) | 80.3 | NR | 0.312 | - |
|  | Mutated | 5/5 (100) | 100 | NR |  |  |
| *FLT3-ITD* | Negative | 36/45 (80.0) | 78.9 | NR | 0.402 | - |
|  | Positive | 3/3 (100.0) | 100.0 | NR |  |  |
| *TP53* | Wild Type | 16/18 (88.9) | 88.9 | NR | 0.230 | - |
|  | Mutated | 7/10 (70.0) | 68.6 | NR |  |  |
| Karyotype | Fav./Int. | 27/31 (87.1) | 86.2 | NR | 0.223 | - |
|  | Poor | 14/19 (73.7) | 72.9 | NR |  |  |
| Therapy Related | No | 29/37 (78.4) | 77.1 | NR | 0.275 | - |
|  | Yes | 12/13 (92.3) | 92.3 | NR |  |  |
| ELN 2017 | Low/Int. | 22/25 (88.0) | 86.6 | NR | 0.286 | - |
|  | High | 19/25 (76.0) | 75.6 | NR |  |  |
| MRD TP1 | Negative | 11/14 (78.6) | 76.2 | NR | 0.732 | - |
|  | Positive | 20/24 (83.3) | 83.3 | NR |  |  |
| AlloBMT  (landmark) | BMT CR1 | 20/20 (100) | 100 | NR | 0.011 | 0.002 |
|  | No BMT CR1 | 21/29 (72.4) | 70.5 | NR |  |  |
